# Recommendations for Effective Intersectoral Collaboration in Health Promotion Interventions: Results from Joint Action CHRODIS-PLUS Work Package 5 Activities

**DOI:** 10.3390/ijerph17186474

**Published:** 2020-09-05

**Authors:** Djoeke van Dale, Lidwien Lemmens, Marieke Hendriksen, Nella Savolainen, Péter Nagy, Edit Marosi, Michela Eigenmann, Ingrid Stegemann, Heather L. Rogers

**Affiliations:** 1 National Institute of Public Health and Environment, 3720 BA Bilthoven, The Netherlands; lidwien.lemmens@rivm.nl (L.L.); marieke.hendriksen@rivm.nl (M.H.); 2 National Institute for Health and Welfare, Fl-100271 Helsinki, Finland; nella.savolainen@thl.fi; 3 National Institute of Oncology, 1122 Budapest, Hungary; peter.nagy@oncol.hu (P.N.); marosi.edit@oncol.hu (E.M.); 4 Foundation IRCCS Neurological Institute “Carlo Besta”, 20133 Milan, Italy; michela.eigenmann@istituto-besta.it; 5 EuroHealthNet, 1000 Brussels, Belgium; i.stegeman@eurohealthnet.eu; 6 Biocruces Bizkaia Health Research Institute, 48903 Barakaldo, Spain; rogersheatherl@gmail.com; 7 Ikerbasque Basque Foundation for Science, 48013 Bilbao, Spain

**Keywords:** chronic diseases, disease prevention, health promotion, intersectoral action for health, intersectoral collaboration

## Abstract

The burden of chronic disease in Europe continues to grow. A major challenge facing national governments is how to tackle the risk factors of sedentary lifestyle, alcohol abuse, smoking, and unhealthy diet. These factors are complex and necessitate intersectoral collaboration to strengthen health promotion, counter-act the social determinants of health, and reduce the prevalence of chronic disease. European countries have diverse intersectoral collaboration to encourage health promotion activities. In the Joint Action CHRODIS-PLUS success factors for intersectoral collaboration within and outside healthcare which strengthen health promotion activities were identified with a mixed method design via a survey of 22 project partners in 14 countries and 2 workshops. In six semi-structured interviews, the mechanisms underlying these success factors were examined. These mechanisms can be very context-specific but do give more insight into how they can be replicated. In this paper, 20 health promotion interventions from national programs in CHRODIS PLUS are explored. This includes community interventions, policy actions, integrated approaches, capacity building, and training activities. The interventions involved collaboration across three to more than six sectors. The conclusion is a set of seven recommendations that are considered to be essential for fostering intersectoral collaboration to improve health-promoting activities.

## 1. Introduction

Chronic diseases are the leading cause of mortality and morbidity in Europe [1,2,3]. One of the strategies to decrease the burden of chronic disease in Europe is tackling the major risk factors such as sedentary lifestyle, alcohol abuse, smoking, unhealthy diet, and cancer [4,5,6,7]. There is much evidence of the value of health promotion to health systems’ performance, outcomes, and sustainability [8,9,10]. Disadvantaged groups, however, are often out of reach of health-promotion activities [4]. Reasons for infrequent uptake include the fragmentation of services and lack of integration within regular care [11].

Another cause of the difficulties to overcome when tackling this problem is the complex nature of chronic diseases [12]. Wider social determinants are underlying causes of unhealthy behavior and the onset of chronic diseases [13]. These social determinants of health are complex, dynamic, and interdependent [14,15]. Given the interdependent nature of the determinants, intersectoral collaboration is necessary. The health sector cannot solve such a complex problem alone [6,9,16,17,18]. Therefore, effective collaboration between different sectors is urgently required to improve health across society [19,20].

For example, concerning impact on areas of life such as the employment sector, recent data from 27 EU member states showed that about one quarter of the working age population (23.5%) had a chronic disease, while 19% reported having long-standing health issues. Work and health are interrelated in many ways. The ageing of the working population combined with the dramatic low employment rates of persons with chronic diseases (PwCDs) is an indicative depiction of this particular relation [21,22]. All mechanisms should champion the importance of strengthening health promotion, preventive services, public health, and social care [23]. This includes engaging partners from other sectors and identifying opportunities for collaboration and seeking synergies to improve health system performance, outcomes, and sustainability.

Since the Ottawa Charter [24] introduced the importance of intersectoral collaboration for health and reduction of health inequalities, numerous studies have been published. A variety of terms and definitions have been used for the collaborative work in healthcare and public health: intersectoral action, intersectoral action for health [25], intersectoral cooperation [26], intersectoral collaboration [27,28], and intersectoral partnerships [29]. The terms are often being used interchangeably. In his review, Dubois et al. searched for a consensual definition for the intersectoral work but could not find such a definition [25]. As a result, the authors constructed their own definition based on the structure: What: *what is the action* (*process, collaboration, coordination*)? Who: *who are the actors conducting the collaboration?*, and Why: *what are the goals or objectives of the action*? Their definition of intersectoral action for health is: *the recognized relationship between a part or parts of the health sector with a part or parts of another sector that has been formed to take action on an issue to achieve health outcomes and intermediate health outcomes in a more effective, efficient, or sustainable way than could be achieved by the health sector acting alone* [25]. This intersectoral collaboration can take place on different levels [30,31]:

- Horizontal collaboration, which occurs between sectors *within* the health sector and *between health and non-health sectors*.
- Vertical collaboration, which occurs between different levels of government, geography, or organization.

In this paper, intersectoral collaboration refers to horizontal collaboration, focusing on collaborations within the healthcare sector and, importantly, between health and non-health sectors. Collaboration with many parties is important for success in health promotion and reduction of health inequalities but is also challenging. It is important to build on what is already known about the important elements for intersectoral collaboration. Danahar et al. [30] identified successful elements for intersectoral collaboration aiming to reduce health inequalities such as shared vision, strong relationship among partners (effective mix), and leadership (advancing purposes, resources, and sustainability of collaboration and efficient structures and processes to do the work). Similar elements are cited when examining collaboration in primary healthcare: enhancing staff satisfaction, defining and selling program goals, increasing professional capacity, establishing flexible legal and structural framework, building trust, promoting collaboration as competency, developing nationals goals through organic participatory processes, aligning structural incentives according to program goals, creating organizational synapses through information technology, and developing innovative monitoring and evaluation schemes [32]. Storm et al. [33] identified five steps as a base for Health in All Policies (HiAP): involvement of the appropriate policy sector in public health, harmonization of objectives, coordinated use of policies and actions by relevant policy sectors, formalized collaboration and experience amongst relevant policy sectors, and favorable contextual factors [33]. Many factors are contextual [32], but some general facilitating elements for intersectoral collaboration can be derived: shared vision of problems to be addressed, strong relationship among partners, mutual and joint benefits/win-win, resources and funding, communication, involvement of community and target group, leadership, capacity building/training, time to build a relationship, and macro-level context (e.g., changes on system level).

The Joint Action CHRODIS PLUS [34] aims to support European countries to improve the prevention of chronic diseases as well as their management, by piloting and implementing innovative approaches that have proven to be successful in other countries or settings. Joint Actions are a type of funding under the third EU Health Programme 2012–2020. They encourage and support cooperation between Member States to improve the health outcomes that benefit their citizens. Across 8 Work Packages, the Joint Action includes 21 pilot implementations and 17 policy dialogues. The pilot projects focus on the following areas: health promotion and primary prevention, an Integrated Multimorbidity Care Model, fostering the quality of care for people with chronic diseases, Information Computer Technology-based patient empowerment, and employment and chronic diseases. Work Package 5, on Health Promotion and Disease Prevention, involves 22 partners from 14 countries. As part of Task 5.3, these partners identified twenty health-promotion interventions involving intersectoral collaboration within and outside healthcare which have strengthened health-promotion activities.

This paper describes the outcomes of CHRODIS PLUS Task 5.3. The aims of this were three-fold: (1) to identify the cross-cutting success factors for intersectoral collaboration identified through the analysis of twenty different European health-promotion and disease-prevention interventions, both in national and community settings, (2) to explore the mechanisms underlying the success factors and their implementation, and (3) to provide recommendations on how to effectively implement these success factors.

## 2. Materials and Methods

**Figure 1:**
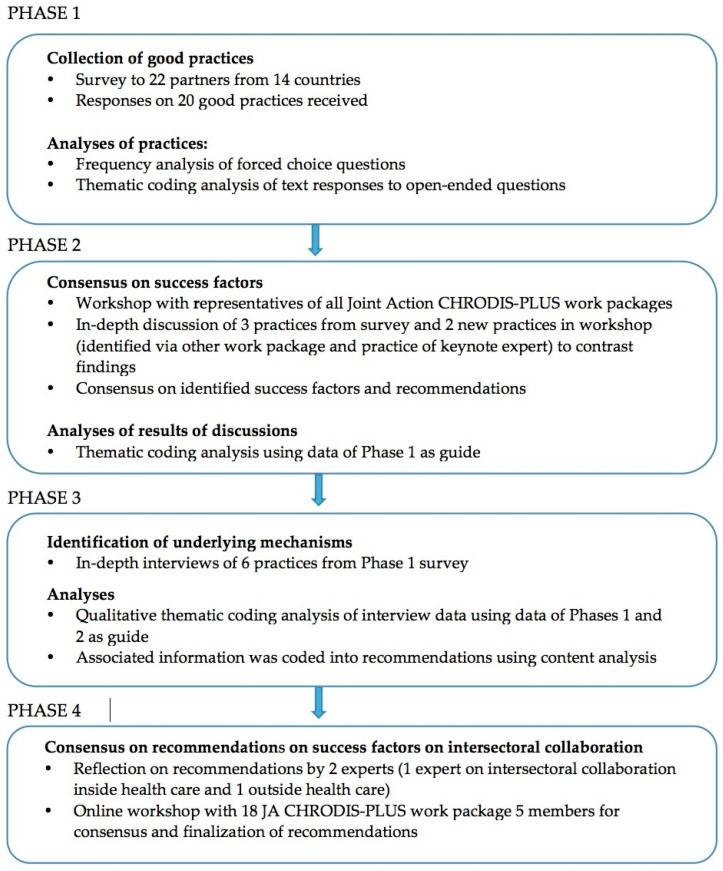
Design of the study: presentation of the data collection and analyses to identify success factors of good practices.

Data for CHRODIS PLUS Task 5.3 were collected and analyzed between April 2018 and May 2020 using a combined grounded theory and case study design implemented across 4 phases of mixed methods data collection. The approach to identifying success factors of intersectoral collaboration in health promotion and disease prevention involved the collection and analysis of ‘good practice’ in this field from across the EU. The four phases of data collection and analysis are presented in Figure 1.

### 2.1. Phase 1: Online Questionnaire Regarding Intersectoral Collaboration in Health-Promotion Practices

An online questionnaire was created based on a review of the literature regarding intersectoral collaboration in health promotion and the criteria for best practices of the Steering Group of Health Promotion and Disease Management (https://ec.europa.eu/health/non_communicable_diseases/steeringgroup_promotionprevention_en). In April 2018, CHRODIS PLUS Work Package 5 Partners (*N* = 22) received a link to the online survey. Each partner was asked to select good health-promotion interventions in their own country that exemplified effective intersectoral collaboration and to fill out the questionnaire for each intervention. Partners were policymakers and professionals working at the Ministry of Health, Public Health Institutes, Health Organizations, Health Service Research Institutes, and a Patient Organization. The questionnaire asked the partners to identify good practices involving horizontal collaboration within healthcare and between the broader health system and other sectors, as well as their enablers and barriers. In the survey, definitions for the key concepts were provided with examples in order to all start from the same conceptual framework (see definition in the introduction), and criteria were explained that needed to be fulfilled to qualify as best practice and examples of best practices and innovative practices were given. Criteria were for example intervention characteristics, effectiveness, transferability, sustainability, and of intersectoral collaboration. In total, data from twenty health-promotion interventions in fourteen countries were received and analyzed.

To analyze the online survey data, a classification score was developed. Points were assigned for a list of key elements that were derived from the literature and adapted from the multiple-choice questions in the questionnaire. Two experts in health promotion coded the textual data obtained from the online survey using a thematic coding procedure. The final coding was agreed by consensus. In cases where consensus could not be reached, a third expert was consulted. The frequency of the scores were shared with CHRODIS Work Package 5 partners, who were asked for feedback and offered suggestions for further analyses (such as more illustrative examples of the good practices).

### 2.2. Phase 2: Workshop Examining Intersectoral Collaboration in Health-Promotion Interventions

In May 2019, CHRODIS PLUS partners from all Work Packages participated in a workshop led by Work Package 5 on intersectoral collaboration in health promotion. Seventy-five professionals from different sectors, including healthcare, employment, patient organizations, public health, and health promotion attended.

A total of five interventions were discussed in the workshop. Three different health-promotion interventions, one disease management practice, and one national program from five different countries were presented to the 75 participants. The participants were representatives from all the work packages (on health promotion, integrated care, quality of care, and employment and chronic diseases) and came from Public Health Organizations, Healthcare Organizations, Health Service Research Institutes, Ministries of Health, Hospitals, Patient Organizations, and Primary Healthcare. Discussions centered on formulating success factors and recommendations for intersectoral collaboration. Three were practices shown in Table 1 (**Healthy Overvecht**, **Vesote Lifestyle Counselling**, **Health Promotion for people at risk of cardiovascular disease and diabetes**) and two practices were not included in Table 1: **Integrated care for people with chronic wounds** (*implementation of a model to integrate care within and outside healthcare for people with chronic wounds. The program entails preventive visits by nurses, new programs, e.g., physical activity for elderly people and for people with type 2 diabetes, in the health promotion centers, and collaboration with social care institutes (Slovenia)* from another CHRODIS PLUS work package to increase interaction among members of the partnership) and the **Prevention of Childhood Obesity** (*additional plan with a mix of interventions to prevent overweight and obesity of children (Hungary)*, key note presentation on a national program presented by the expert on intersectoral collaboration on the national level in Hungary). The additional practices were used to contrast initial findings.

At the workshop, an experienced facilitator familiar with the results of the Phase 1 online survey led a discussion about the role of intersectoral collaboration in the interventions. Success factors enabling effective intersectoral collaboration were identified during the small group discussions focusing on the five above-mentioned examples. The facilitator helped the groups identify the success factors and to formulate recommendations to improve intersectoral collaboration. The data collected during the workshop on success factors and recommendations was coded using thematic coding by two experienced experts in health promotion using the Phase 1 data as a guide.

### 2.3. Phase 3: Semi-Structured Interviews to Provide In-Depth Insight into Intersectoral Collaboration in Health-Promotion Interventions

A semi-structured interview guide was developed in order to further explore strategies to carry out intersectoral collaboration in health-promotion interventions effectively. The aim of the interview was to identify the underlying mechanisms of the success factors, in other words, to understand how the success factors were accomplished. Enabling and hindering factors were examined, as well as ways to overcome barriers that arose during the intersectoral collaboration process.

From the original 20 practices in Table 1, six health-promotion interventions in four countries were identified for in-depth case study analysis representing different types of programs (national and community) and topics (overweight, smoking cessation, healthy lifestyle, and integrated medical and social care: Young People at Healthy Weight (JOGG, Netherlands), Healthy Overvecht (Netherlands), Vesote Lifestyle Counselling (Finland), Tobacco Cessation Services (Finland), Smoke-free Hungary (Hungary) Lombardy Workplace Health Promotion Network (Italy)). See Table 1 for specific details on these interventions. Three of the six practices were also discussed in Phase 2. Interviewees were program leaders of the practices.

CHRODIS PLUS Work Package 5 Partners interviewed the professional most familiar with the health-promotion intervention in their native language. After the conclusion of the interview, an English summary of the data collected was provided. Each interview was analyzed by two researchers using thematic content analysis. In case of any discrepancies in coding, a third researcher was used to reach consensus. Using the list of factors identified in the Phase 1 questionnaire, associated information was coded into recommendations using content analysis. In case of doubt, the assistance of the third investigator was used. The final coding was established by consensus. This coding was checked and agreed upon by Work Package 5 partners.

### 2.4. Phase 4: Development of Recommendations for Successful Intersectoral Collaboration

The coded recommendations from the interviews and workshop were combined. Recommendations were clustered by theme by three researchers and consensus was reached on these clusters. Then, these clusters were sent to Work Package 5 partners for feedback. Consensus was reached on six recommendations in which both the rationale (Why?) and the actions/steps (How?) could be detailed and illustrated with an example from the Phase 2 semi-structured interviews. These draft recommendations were then presented to CHRODIS PLUS Work Package 5 partners at a second workshop. Because of the outbreak of COVID-19, the planned in-person workshop was not feasible and instead an online meeting was organized to finalize the recommendations. In preparation for this workshop, an online questionnaire was sent to all registered participants (22 organizations from 14 countries). They were asked whether they thought the draft recommendations (including the Why and How) were feasible or needed adaptations. Additionally, prior to the workshop, two experts, one in integrated healthcare and one in intersectoral collaboration outside the healthcare sector, were asked to reflect on the draft recommendations. The feedback from partners and experts were summarized and adaptations were made to the recommendations accordingly, which resulted in the addition of a seventh recommendation. The online workshop was held in May 2020 with 18 participants working as policymakers, researchers, or practitioners at the Ministry of Health, Public Health Institutes, and Health Organizations in attendance. An experienced facilitator led the discussion of participant feedback and helped the group reach consensus on the final wording for all seven recommendations.

## 3. Results

### 3.1. Descriptive Results Regarding Intersectoral Collaboration in Health-Promotion Interventions

The Phase 1 online survey resulted in data from twenty health-promotion interventions in fourteen countries. Table 1 describes each intervention, its type and duration, and its aim(s) and target population. In short, most of the interventions (*N* = 17) focused on both health promotion and specific disease prevention, with only three interventions solely addressing health promotion. The health-promotion interventions aimed to improve the unhealthy lifestyle factors such as unhealthy diet, smoking, sedentary lifestyle, alcohol misuse, and stress prevention. Improving health literacy and reduction of health inequalities were also targeted. Most of the interventions (*N* = 16) were national programs and long-lasting, with a typical duration of more than five years. These programs consisted of a mix of discrete health-promotion strategies or practices such as community interventions, policy actions, integrated approaches, and capacity building and/or training. Since most program interventions consisted of a mix of interventions, the degree of collaboration was considerable. Half of the interventions worked together with three or more disciplines within healthcare. Eight collaborated with more than six sectors outside the healthcare sector and seven engaged in intersectoral collaboration with three to five sectors outside the healthcare sector. Four interventions collaborated with two other sectors. One intervention did not specify this information about collaborating disciplines or sectors. Because of the wide range of practices, there was also a wide range of collaborating parties, such as ministries (Health, Education, Family Youth and Social Welfare, Social sector, Employment, etc.) at the national level, private organizations such as food industries, (primary) healthcare and public health organizations, and patient organizations such as a Lung Foundation or Diabetes Association or senior clubs, local authorities, hospitals, schools, public health institutes, schools of public health, etc.

The size and specificity of target populations varied considerably across interventions. Some interventions targeted people from different age groups (e.g., infants, children, youth, adults, older adults) and/or their carers (e.g., parents, formal or informal caregivers). Many interventions sought to reach vulnerable or minority groups, such as people with low socio-economic status, immigrants, individuals with low health literacy, and those with high health risks (e.g., history of mental disorders and/or substance abuse, sleep problems, physically inactive or overweight, medical risk factors for specific diseases). To reach the target populations, interventions often targeted different groups of professionals, such as employers, educators, health and social professionals, and policy makers.

The degree of intersectoral collaboration was often related to the scope of the intervention (several lifestyle topics), and the target population to be reached by the health-promotion intervention in this sample, meeting good practice criteria. When the program aimed to reach a large range of the population and targeted to improve lifestyle, high intersectoral collaboration (e.g., more than six sectors and/or three disciplines) tended to be present. To reach the whole population and to have an impact on the health behavior and outcomes, it is necessary to involve different intermediate groups and policymakers/departments and organize a mix of interventions in different settings. That requires intersectoral collaboration. The Andalusion Strategy of Local Action in Health, for instance, was highly intersectoral because they targeted the whole population and the intermediate groups such as private companies (youth) healthcare, schools, sports, employers, municipal health services, and welfare. In contrast, interventions carried out in a specific setting collaborated with fewer sectors, although high multidisciplinarity was achieved. For example, Iceland’s Program of Coordinated Action and Strategy of Health Promotion in School Healthcare worked with two sectors and three disciplines. In general, programs aiming to reduce health inequalities tended to collaborate with six sectors or more. However, it is important to recognize that only good practices were examined. It is possible that health-promotion and disease-prevention programs that aim to target a large range of the population and do not effectively achieve intersectoral collaboration may not have led to good outcomes/results, and therefore, they would not have been included in the sample of good practices studied. Further research on the relationship between reach of a program and degree of intersectoral collaboration is warranted.

A section of the online questionnaire focused on barriers and enablers regarding intersectoral collaboration. Most respondents reported that using a framework to implement the health-promotion intervention and as a base for intersectoral collaboration was a key enabler. Fifteen of the twenty interventions used a framework and these frameworks varied widely and served different functions. Some used legal frameworks (e.g., European, national, or local laws), whereas others used a national plan as a logic model [35] with programs, project, and activities or the strategy of the practice (e.g., strategy for social inclusion of Roma’s). Other respondents highlighted the usefulness of frameworks they developed to organize the collaboration and foster communication, such as a national platform set up by the Ministry of Health and several other ministries, a membership organization, or specific networking methods. Finally, respondents valued frameworks that described how to carry out the work involved, such as the four-domain model (body, mind, social, and relations/network) to provide care in a uniform way (Healthy Overvecht, the Netherlands) and reference manuals, such as “How to become a healthy workplace” (Lombardy Workplace Network, Italy).

Capacity and funding are linked with the sustainability of the intervention. Intervention funding period was a proxy for the duration in the questionnaire. Nine interventions had a funding period of more than four years, seven with cycles lasting two years, and two with funding cycles of less than two years (yearly, for example). Data on duration and funding cycles was missing for two interventions. Interventions that were smaller in scope, such as Smoking Cessation in Mental Healthcare (Finland), were more likely to have short funding periods and this was raised as a barrier in the evaluation section of the questionnaire. However, some interventions, such as Hygiene Week (Denmark), lacked a longer-term funding cycle, but has been carried out for the past ten years.

### 3.2. Key Enablers and Barriers of Intersectoral Collaboration

In the survey, respondents were asked what they considered the most important and key success factors for successful collaboration. In Table 2, the Top 10 most frequently named elements are shown. The factors ‘*a shared vision of the problem to be addressed and the success of the collaboration*’, ‘*communication*’, and ‘*a win-win for partners in the collaboration* (*mutual and joint benefits*)’ were mentioned most frequently.

The barriers of successful intersectoral collaboration described in the survey results tended to be a lack of the enablers identified in Table 2. The critical barriers most commonly reported by CHRODIS Work Package Partners in the online survey were: inadequate support and uptake in policies (*n* = 6), no shared vision (*n* = 4), insufficient personnel, personnel lacking time or capacity (*n* = 4), insufficient funding (*n* = 3), and poor trust among collaboration partners (*n* = 3).

### 3.3. Drafting Recommendations

To be able to draw up recommendations, the key enablers of successful intersectoral collaboration identified in the online survey were first verified by CHRODIS PLUS partners from various sectors who attended the Phase 2 workshop. Additional success factors were raised during the workshop discussions, including the use of champions and the use of external policy directives (e.g., Sustainable Development Goals) to align intervention objectives. Next, semi-structured interviews were conducted in Phase 3 to achieve an in-depth examination of the key enablers in six interventions. The in-depth interviews allowed enablers for successful intersectoral collaboration to be probed in ways that elicited specific strategies to achieve or “bring to life” that particular enabler.

The enablers were then clustered by theme and translated into draft recommendations. During analysis, a hierarchy was created. Specific recommendations identified to achieve certain enablers were also steps to reach another more generic recommendation on a higher abstract level. For example, the recommendations *Check existing collaboration networks and collaboration partners* and *Involve community/target group from the start* were placed under the ‘how’ of the more generic recommendation to *Engage all relevant stakeholders.* After reaching consensus among coders about the clustering, the hierarchical order of the recommendations, and illustrative examples, the six main recommendations were formulated. Each recommendation described a rationale for the recommendation and detailed steps to achieve the recommendation.

The draft recommendations were sent to experts and Work Package 5 partners for feedback. After this consultation, a seventh recommendation was added: *Align with key policies and search for political support*. Both experts and one partner considered this recommendation to be one of the most important recommendations and worthy of its own recommendation. Originally, it had been included as a ‘how’ of another recommendation. An online workshop was held to agree upon the final wording and presentation of the recommendations. An experienced facilitator guided the discussion and, with minor changes, all meeting participants from Work Package 5 approved the recommendations as part of the Task 5.3 final report. These final recommendations for effective intersectoral collaboration are presented in Table 3.

## 4. Discussion

The success factors of intersectoral collaboration of a wide range of health-promotion and disease-prevention interventions from fourteen countries all over Europe have been explored and seven recommendations for effective implementation of intersectoral collaboration have been detailed. Of the fourteen countries taking part in Work Package 5 of CHRODIS PLUS on Health Promotion and Disease Prevention, six Eastern European countries (e.g., Bulgaria, Croatia, Hungary, Poland, Lithuania and Serbia,) contributed, in addition to Western European Countries. This is a strength of the present recommendations on intersectoral collaboration, as data on health promotion in Eastern European countries tends to be less prominent in the scientific literature. The interventions explored were similar in type, being national programs combined with local programs. All interventions had similar barriers and enablers to intersectoral collaboration.

The recommendations that resulted from Task 5.3 are in line with the literature [29,30,32,36,37,38]. Corbin et al. [36] suggests nine core elements that constitute positive partnership processes. The final seven recommendations and implementation strategies for effective intersectoral collaboration incorporate almost all aspects of Corbin’s nine core elements (*mission, resources, leadership, communication, roles/structure, input interaction, maintenance and production tasks, context and evaluation of additive results, synergy and antagony*). In the present study, evaluation and context are not stated as separate recommendations, although evaluation is part of the planned approach. Context however is not part of the present recommendations and needs more attention in the process of collaboration.

Furthermore, recent work of INHERIT 2019 (INter-sectoral Health and Environment Research for InnovaTion), in which triple win cases (identifying ways of living, moving, and consuming that protects the environment and improve health and well-being) were collected, showed similar results [26]. They defined ten elements of good practice on intersectoral collaboration: *Develop a triple win mindset, establish international, national or local priorities, embed initiatives in international, national, or local priorities, bring together sectors around a common interest, engage people and communities of interest for co-creation, ensure that initiatives are inclusive, explore effective or new ways to secure long-term funding, integrate ways of evaluating initiatives, identify strengths and positive feedback loops, and embed the triple win from an early age* [26]. The INHERIT project addressed the reduction of health inequalities as one of its main targets, hence the recommendation about ensuring that initiatives are inclusive. Although many of the interventions evaluated in Task 5.3 addressed health inequalities, and inclusiveness is part of Recommendation 3, creating an effective mix of partners’, this aspect was not highlighted specifically in the final recommendations. This omission was due to the fact that the focus of the present study was on strengthening health-promotion activities to prevent chronic diseases and not specifically on reducing health inequalities. Future efforts to facilitate intersectoral collaboration should evaluate the role of inclusiveness and, if determined valuable, recommendations for how to achieve it.

Placing this study in the context of overall program implementation [39], the focus was on one specific barrier—intersectoral collaboration. The results examined barriers and facilitators for this successful collaboration. The participants described barriers that tended to be the inverse of the enabling factors identified. Other studies also mention this duality [30,38] or present only facilitating factors [29,36]. Leenaars et al. [38] presented several of our enabling factors as factors that facilitate the collaboration or whose absence hindered it. Common key factors mentioned in the program implementation literature as barriers are partly in line with our findings that are specific to intersectoral collaboration, including financial and time constraints, the influence of the wider and political context, workplace structuring, and staff turnover [30].

The overall aim of CHRODIS Task 5.3 was to identify interventions in which intersectoral collaboration was important for effective health-promotion activities. Horizontal intersectoral collaboration within the healthcare sector and collaboration outside the healthcare sector was the specific focus. Very few interventions addressed intersectoral collaboration within the healthcare sector, as there is more and more debate in the European countries about the necessity for a change from focus on care towards more prevention and health promotion because of the contribution to decreasing the costs. This can be explained by the fact that since the Ottawa Charter, the collaboration with sectors outside the healthcare sector is seen as one of the most promising actions to strengthen health promotion. Further study of intersectoral collaboration within healthcare is warranted, as the seven recommendations may need more elaboration for this specific sub-type of intersectoral collaboration which was under-represented in the sampling of the interventions.

The value of a planned and systemic approach to implementation of the health-promotion intervention was identified as a recommendation for successful intersectoral collaboration. This coincides with ample literature indicating that the use of a framework is an important basic element for collaboration [32]. Dubois defined several frameworks supporting collaboration [25]. A framework permits a common understanding of an approach and provides a structure to evaluate how different factors (e.g., conditions for success) connect with each other. Although several frameworks exist, no one framework emerges from the literature as a gold standard [25]. In addition, frameworks are used for different functions. Some are used for research aims (Bergen model [40]), to list conditions for success [41], to identify potential key mechanisms [42], or to develop a comprehensive list of coordinated action [43]. Most interventions examined as part of Task 5.3 used a framework, but no specific frameworks were identified by the participants as valuable to guide the intersectoral collaboration specifically.

Leadership was another key enabling factor and recommendation for intersectoral collaboration. Given that a large number of national health-promotion interventions with regional components were explored in CHRODIS PLUS Task 5.3, the benefits of national leadership were emphasized by participants. Guglielmin et al. [44] distinguished between local and national leadership, but this distinction did not emerge from the data collected in this study [44]. Moreover, regardless of the level of action, Corbin et al. [36] suggests that there are several forms of leadership, but all leaders must have the ability to inspire trust, instill confidence, be inclusive of diverse partners, and be collaborative and transparent in the decision-making process.

Building upon existing structures and collaborations was mentioned as an important factor. As health challenges increase in complexity nowadays, multi-level and multi-disciplinary health-promotion interventions will become the norm. In this regard, capitalization on existing networks will likely become increasingly important over time. Improving and strengthening existing health and decreasing health inequalities is complex, and permanent collaboration structures are needed to permit timely response to these challenges. While the focus of health promotion programs may be different (e.g., overweight, fall prevention, physical activity), the approaches that must be taken should, in general, be similar. Successful implementation of these programs requires an established prevention infrastructure, independent of the topic, which is spearheaded by the local or regional government. In addition, they should cooperate with other public organizations as well as with private enterprises and non-profit organizations. Future research is needed to identify good practices regarding the creation and maintenance of these kinds of permanent structures or networks.

### Limitations

To identify the good practices and the enabling factors for intersectoral collaboration, an online questionnaire was sent, and two workshops were organized. With this approach, it was expected to find more and diverse practices. During analyses and the discussions with partners in the first workshop, it appeared information was missing about *how* to achieve the success factors (and recommendations). That is, the question remained as to which strategies or steps are needed to implement the recommendation to achieve successful intersectoral collaboration? Therefore, six in-depth interviews were performed additionally with the practice owners of different types of programs (national/local and diverse topics) to gain more insight into the elements and processes to achieve the success factors in the collaboration.

Although not specifically addressed in the seven recommendations, the role of context continues to be an important challenge, as context influences the barriers and enablers and the mechanisms through which successful implementation of a recommendation is achieved. Moreover, successful implementation of one recommendation creates contextual changes that make the probability of achieving others more likely. The data collected did not allow for a determination of the necessary and/or sufficient criteria for successful intersectoral collaboration. It is not known if all of the recommendations must be implemented or if certain ones are more essential than others. In a recently published article from CHRODIS-PLUS on health-promotion interventions, context is considered to be one of the key factors for successful implementation [45], but it is challenging to study. To adequately and appropriately address contextual factors, a realist evaluation method can be used [46,47,48]. Steenkamer and colleagues wrote a review on integrated care, healthcare, social care, and wider public services [49], in which they present eight guiding principles with insight into strategies (the HOW), the necessary context (the example, see Table S2 for the elaborated version of the recommendations), and the extracted theory that underlie the recommendation (the WHY). Only one specific context (the example) per recommendation is described in this study. Had the realist evaluation method been used, much more information on inhibiting and enabling contexts would have been gathered, which would have provided more supporting evidence for underlying mechanisms of action which are crucial for the successful implementation of the recommendations. We recommend that program teams use a realist approach to tailor the recommendations that describe their particular context.

Lastly, the interventions sampled were mainly long-term national programs or initiatives with a regional component, which were long-lasting or already coming to an end. Having sufficient data on effectiveness of the intervention was one of the “good practice” criteria. As discussed prior, the interventions primarily involved intersectoral collaboration between health and non-health sectors. Thus, these recommendations on successful intersectoral collaboration may not generalize to all types of health-promotion and disease-prevention programs. Furthermore, the collected data on the value of these recommendations is retrospective. Although a prospective evaluation of the usefulness of these recommendations for successful intersectoral collaboration was planned, its implementation had to be postponed due to the COVID-19 outbreak.

## 5. Conclusions

In the framework of the Joint Action CHRODIS PLUS, Task 5.3 examined twenty health-promotion and disease-prevention programs from all over Europe. Experiences associated with successful intersectoral collaboration were synthesized to determine cross-cutting barriers and enablers and generate a set of seven recommendations. Each recommendation includes concrete steps to implement the recommendation and was found, in general, to be in line with the literature. The recommendations included: connecting with existing policies and advocating for political support, defining a shared vision, creating an effective mix of different partners, encouraging effective leadership, keeping collaboration partners engaged, using a planned systematic approach, and ensuring sufficient resources to sustain the collaboration. These recommendations and their implementation strategies will be used by CHRODIS PLUS partners to enhance intersectoral collaboration and consequently, strengthen health-promotion activities in intervention programs across Europe.

## Figures and Tables

**Table 1:** Overview of good practices on health promotion (see also Table S1).

	Practice	Topic and Themes	Type	Target Group	Collaboration
1	Young people at a healthy weight Netherlands 2010–ongoing Interview ^1^	Health promotion: overweight, physical activity, reduction of health inequalities, and healthy nutrition	National program Community intervention Policy action Integrated approach Training and capacity building	Children aged 0–19 years and intermediary groups (e.g., teachers, sport coaches, business partners, health professionals)	>6 sectors. 3 disciplines
2	Healthy Overvecht (Neighborhood in the city of Utrecht): Integrated medical and social basic care Netherlands 2006-ongoing Workshop ^2^ Interview ^1^	Health promotion and disease prevention: lifestyle factors, health literacy, wellbeing, reduction of health inequalities, and social problems	Community intervention Integrated approach	All inhabitants of the neighborhood, most having a low social economic status.	3–5 sectors >6 disciplines
3	Prevention of cardiovascular system and respiratory system diseases—using Comprehensive Geriatric Assessment Poland 2018–2019	Health promotion and disease prevention: wellbeing, prevention of diseases of the cardiovascular and respiratory system, and reducing the health risks of older people	Policy action Regional program (local program)	People aged 60+ and their carers.	3–5 sectors 4–5 disciplines
4	National Health Plan/Plano Nacional de Saúde Portugal 2012–2020	Health promotion and disease prevention: overweight, physical activity, alcohol prevention, smoking, self-management, health literacy, wellbeing, reduction of health inequalities	National program Community intervention Policy action Integrated approach	General Portuguese population and health Professionals	More than 6 sectors >6 disciplines
5	Tobacco Cessation Services for Patients with Mental Health Disorders and Substance Abuse Finland 2017–2018 Interview ^1^	Health promotion and disease prevention: smoking	National program Health Service Delivery Policy action Training, capacity building Online intervention program	11 hospital districts are involved: a multi-professional tobacco cessation expert group has been established in all hospital districts	2 sectors 3 disciplines
6	Healthy Aveiro Program Portugal 2013–ongoing	Health promotion and disease prevention: health literacy, reduction of health inequalities	Community intervention Integrated approach	Groups experiencing socioeconomic vulnerability, adverse health conditions, and/or have low health literacy.	3–5 sectors 3 disciplines
7	Health-promotion program for people with risk of cardiovascular disease and diabetes Lithuania 2015–ongoing Workshop ^2^	Health promotion and disease prevention: overweight, physical activity, alcohol prevention, smoking, self-management, health literacy, and wellbeing	National program	(1) Persons at the age of 40–65 years selected for Prevention Program CVD (2) Adults, who are assigned to persons at risk.	3–5 sectors
8	Walking on the path of wellbeing Italia 2012–2014	Health promotion and disease prevention, physical activity and wellbeing	Integrated approach	People with sedentary behavior, in particular patients with chronic diseases and those over 65 years old.	>6 sectors 3 disciplines
9	VESOTE project Finland 01/2017–12/2018 Workshop ^2^ Interview ^1^	Health promotion and disease prevention: overweight, physical activity, heathy food, and better sleep without medication	National program Health Service Delivery Integrated approach Training, capacity building	Physically inactive persons, persons suffering sleep problems, diabetics, coronary patients, overweight and obese patients	>6 sectors >6 disciplines
10	The Strength in Old Age Program Finland 2005–ongoing	Health promotion: physical activity, health literacy, wellbeing, and reduction of health inequalities	National program Policy action Integrated approach Training, capacity building Online intervention program	Community-living 75+ persons with decreased mobility and intersectoral collaboration group	3–5 sectors 3 disciplines
11	The Hygiene Week Denmark 2009–2019 (every year)	Health promotion and disease prevention: self-management and health literacy	National program Community intervention Health Service Delivery Policy action Integrated approach Media campaign	General population	3–5 sectors 4–5 disciplines
12	The Andalusian Strategy of Local Action in Health Spain 2008–ongoing	Health promotion and disease prevention: overweight, physical activity, alcohol prevention, smoking, self-management, health literacy, wellbeing, and reduction of health inequalities, healthy aging, accident prevention, sexual and reproductive health, violence prevention, gender issues, environmental health, urban health	Community intervention Policy action Integrated approach Training, capacity building Intersectoral approach Participation Governance	General population of 778 municipalities of the Autonomous Community of Andalusia (Spain)	>6 sectors 4–5 disciplines
13	Gaining Health—making healthy choices Italy 2007–ongoing	Health promotion and disease prevention: overweight, physical activity, alcohol prevention, smoking, wellbeing, reduction of health inequalities, and nutrition	National program Community intervention Policy action Integrated approach	Life course approach: addressing all ages and all public and private environments	>6 sectors 3 disciplines
14	Living Healthy Croatia: 2016–2022	Health promotion and disease prevention: overweight, physical activity, alcohol prevention, smoking, health literacy, wellbeing, and mental health/child depression	National program Community intervention Integrated approach Training, capacity building	Life course approach: with a special focus on persons with heightened behavioral and biomedical risk factors	>6 sectors 3 disciplines
15	Coordinated strategy and action in health promotion for school healthcare Iceland 2006–ongoing	Health promotion and disease prevention: overweight, physical activity, alcohol prevention, smoking, self-management, health literacy, wellbeing, and reduction of health inequalities	National program	School-aged children (6–15 years old) as well as school nurses, teachers, and other school personnel.	2 sectors 3 disciplines
16	The process towards a smoke-free Hungary—Tobacco control in practice Hungary 2011–ongoing Interview ^1^	Health promotion and disease prevention: smoking	National program Policy action Case study	Children, young adults and adults.	2 sectors
17	Living with Diabetes: Education and Weight Management Malta 2015–ongoing	Health promotion and disease prevention: overweight, physical activity, self-management, and health literacy	National program	Overweight and obese patients who have type 2 diabetes.	-
18	Roma health mediators Serbia 2009–ongoing	Health promotion and disease prevention: health literacy, well-being, and reduction of health inequalities	National program Community intervention Health Service Delivery Training, capacity building	Roma ethnic minority population in Serbia.	3–5 sectors and <2 disciplines
19	National Program for Prevention of NCDs (noncommunicable diseases) Bulgaria 2014–2020	Health Promotion and disease prevention: overweight, physical activity, alcohol prevention, smoking, self-management, health literacy, and main NCDs	National program Community intervention Health Service Delivery Policy action Integrated approach Training, capacity building	Life course approach: but especially focuses on women of reproductive age, workplaces, health professionals, and individuals with low socioeconomic status	>6 sectors
20	The Lombardy Workplace Health Promotion (WHP) Network Italy 2014–ongoing Interview ^1^	Health promotion and disease prevention: physical activity, alcohol prevention, smoking, food, work–life balance, and road safety	Integrated approach Regional program	All company workers are involved (young adults, adults, male, and female)	2 sectors

^1^ This practice has been interviewed for more in-depth information. ^2^ This practice has been presented during the workshop.

**Table 2:** Top 10 intersectoral collaboration enablers according to the CHRODIS-PLUS Work Package 5 Partner Online Survey.

Key Enablers	Frequency of Good Practices
A shared vision of the problem to be addressed and the successes of the collaboration	13
Communication	13
A win-win for partners in the collaboration (mutual and joint benefits)	11
There is uptake in structural processes (clarity about roles and responsibilities, availability of protocol)	9
Macro-level context is taken into account (changes on system level)	8
Capacity, e.g., enough personnel, personnel have enough time, and qualified personnel	7
Trust between collaboration partners (e.g., trust between health sector and welfare sector)	7
Recruitment of diverse partners (effective mix)	6
The intervention has a strong leadership in advancing shared purposes	6
There is support and uptake in policies	6

**Table 3:** Seven recommendations for effective intersectoral collaboration with the rationale and steps to implement the recommendation (see Table S2 for a detailed table, with descriptions of the interventions that illustrate the implementation of the recommendation in practice).

1	Connect collaborationgoals with existing key policies, while actively advocating for political support
	Why? Political support is a prerequisite to get resources allocated for the implementation and the sustainability of health-promotion programs. In order to gain political support, collaboration goals should be aligned with key policies How? Ensure that the planning documents contain the references to important policies Align with health system goals Make use of existing system changes
2	Define a shared vision of the problem to be solved aligned with organizational goals
	Why? Commitment of all partners is crucial for successful collaboration. Agreeing on the problem to be solved and defining a shared vision of how to solve the problem helps to create this commitment and results. Furthermore, such a discussion allows professionals from different organizations, and possibly sectors, to develop a common language to talk about the main issues and potential solutions. How? Appeal to a shared sense of urgency to solve a problem or to shared interests Agree on intersectoral collaboration as one of the solutions of the problem Achieve actual mutual understanding of norms, values, and roles and create trust Use a visionary leader who is accepted by all parties Engage an experienced facilitator/coordinator
3	Create an effective mix of different partners with diverse backgrounds and skills
	Why? To be able to reach the target group effectively, all relevant parties that could influence the health behavior of the target group should be involved in the collaboration. How? Identify and involve strategic partners with access to and/or experiences with the target group Capitalize on existing partners and available collaboration networks Allow ample time for building new relationships Involve representatives of the target group and community from the start Use standard methods for stakeholder mapping
4	Build bridges between sectors and disciplines through effective leadership
	Why? Leadership is essential and closely tied to strong working relationships and a transparent process for collaboration. Effective leadership fosters trust and good working relationships between collaboration partners. How? Identify a local champion who can be the leader or can support the leader Use different types of leaders or leadership for different phases of the collaboration Recruit a dedicated person with leaderships and coordination qualities: who understands the language of ‘others’ with good project and process management skills who uses information systems and technologies to ensure effective communication and information exchange
5	Keep collaboration partners in all sectors engaged
	Why? Crucial for the success of the collaboration is keeping the partners engaged by informing, motivating, and entrusting them, thus sustaining commitment of all partners. How? Formalize the collaboration by making clear agreements about roles and responsibilities of the partners Create a win-win situation for partners in the collaboration (mutual and joint benefits) Form designated communication liaisons, e.g., to provide information to participants of the collaboration, arrange meetings, manage a website, and/or create regular newsletters Give professionals ownership, via a bottom-up approach Motivate the professionals involved, e.g., by offering feedback on progress towards shared vision Celebrate smaller short-term advancements while aiming for long-term, sustainable success Organize face-to-face meetings when possible to allow people from different sectors and disciplines to get to know each other also on an informal and personal level
6	Use a planned/systematic approach suitable for all partners
	Why? Using a systematic approach based on scientific evidence and on experiences from the past will improve the implementation of the collaboration in each sector. Moreover, this systematic approach should allow all partners to combine their health-promotion efforts and enhance the effectiveness of the program. How? Identify a theoretical framework or model that can be used by different sectors Identify a theoretical framework or model that can be adapted to local context Strengthen the collaboration as iterative and adaptive processes Share and learn from experiences Involve experts and others with experience in similar efforts Replicate, and adapt if necessary, best practices that have been shown to result in successful outcomes
7	Ensure there are sufficient resources to sustain the collaboration
	Why? To establish a sustainable collaboration, it is important that resources, such as dedicated time, qualified personnel, and funding, are and remain available. The distribution of these resources should be transparent and fair to all partners. How? Describe needed and obtained resources to facilitate a transparent distribution among the partners Allocate (working hours of) personnel to collaboration Provide training to managers and professionals Acquire or build upon structural resources (e.g., human resources or funding) Communicate about the cost-saving or effective results

## Supplementary Materials

The following are available online at https://www.mdpi.com/1660-4601/17/18/6474/s1, Table S1: Overview of the selected good practices on Health Promotion, Table S2. Seven recommendations for Intersectoral Collaboration with the rationale and steps to implement the recommendation and examples of the practices which show the recommendation in practice.
