# Supplementary material for: Recommendations for Effective Intersectoral Collaboration in Health Promotion Interventions: Results from Joint Action CHRODIS-PLUS Work Package 5 Activities
Source: Int J Environ Res Public Health. 2020 Sep 5;17(18):6474. doi: 10.3390/ijerph17186474 (PMC7557573; doi:10.3390/ijerph17186474)
Supplement: Supplementary file 1 [file ijerph-17-06474-s001.pdf]

International Journal of
Environmental Research and Public Health

Supplementary Materials: Recommendations for Effective Intersectoral Collaboration in Health Promotion Interventions: Results from Joint Action CHRODIS-PLUS Work Package 5 Activities

Table S1: Overview of the selected good practices on Health Promotion

	Practice	Aim	Topic and Themes	Type	Target Group	Collaboration	Evaluation
1	Young people at a healthy weight / Netherlands: NL1<br>2010–ongoing<br>*Interview*[1]	To reverse the increasing trend of young people with overweight /obesity in the Netherlands through the JOGG themes: water, fruit and vegetables consumption and physical activity	Health promotion: overweight, physical activity, reduction of health inequalities and healthy nutrition	• National program<br>• Community intervention<br>• Policy action<br>• Integrated approach<br>• Training and capacity building	Children aged 0-19 years and intermediary groups (e.g. teachers, sport coaches, business partners, health professionals)	>6 sectors<br>3 disciplines	Process and outcome
2	Healthy Overvecht: Integrated medical and social basic care Netherlands: NL2<br>2006–ongoing<br>*Workshop*[2]<br>*Interview*[1]	To make the work of health professionals more sustainable through shifting focus from disease treatment and care to promoting healthy behaviour in Overvecht.	Health promotion and disease prevention: lifestyle factors, health literacy, wellbeing, reduction of health inequalities and social problems	• Community intervention<br>• Integrated approach	All inhabitants of the neighbourhood, most having a low social economic status.	3-5 sectors<br>>6 disciplines	Process and outcome
3	Prevention of cardiovascular system and respiratory system diseases and Comprehensive Geriatric Assessment (CGA) Poland: PL1<br>2018–2019	To increase the effectiveness of recognition and monitoring of cardiovascular system and lung obstructive diseases among elderly people using extended Comprehensive Geriatric Assessment.	Health promotion and disease prevention: wellbeing, prevention of diseases of the cardiovascular and respiratory system and reducing the health risks of older people	• Policy action<br>• Regional program (local program)	People aged 60+ and their carers.	3-5 sectors<br>4-5 disciplines	Process

	Practice	Aim	Topic and Themes	Type	Target Group	Collaboration	Evaluation
4	National Health Plan / Plano Nacional de Saúde Portugal: PT1 2012–2020	To increase health promotion and protection, disease prevention and control, intersectoral collaboration, citizen empowerment, promotion of healthy environments, dissemination and implementation of good practices and strengthening of global health.	Health promotion and disease prevention: overweight, physical activity, alcohol prevention, smoking, self-management, health literacy, wellbeing, reduction of health inequalities	• National program<br>• Community intervention<br>• Policy action<br>• Integrated approach	General Portuguese population and health Professionals	More than 6 sectors >6 disciplines	Process and outcome
5	Tobacco Cessation Services for Patients with Mental Health Disorders and Substance Abuse Finland: FI1 2017–2018 *Interview*[1]	To provide patients with mental health or substance abuse patients with better physical health through adequate tobacco cessation services.	Health promotion and disease prevention: smoking	• National program<br>• Health Service Delivery<br>• Policy action<br>• Training, capacity building<br>• Online intervention program	11 hospital districts are involved: a multi-professional tobacco cessation expert group has been established in all hospital districts	2 sectors 3 disciplines	Process, outcome and participatory
6	Healthy Aveiro Programme Portugal: PT2 2013–ongoing	To address unhealthy conditions, to prevent chronic diseases, to enrich the skill set of professionals dealing with multisectoral interventions, and to develop organisational models to implement this strategy.	Health promotion and disease prevention: health literacy, reduction of health inequalities	• Community intervention<br>• Integrated approach	Groups experiencing socioeconomic vulnerability, adverse health conditions, and/or have low health literacy.	3-5 sectors 3 disciplines	Process
7	Health promotion program for people with risk of cardiovascular disease and diabetes	To implement the prevention program in primary care centres and develop the collaboration between primary care centres, community and	Health promotion and disease prevention: overweight, physical activity, alcohol prevention, smoking,	• National program	1) Persons at the age of 40-65 years who participate in the "Program for the Selection and Prevention Measures	3-5 sectors -	Process and outcome

International Journal of
Environmental Research and Public Health

	Practice	Aim	Topic and Themes	Type	Target Group	Collaboration	Evaluation
	Lithuania: LT1<br>2015–ongoing<br>*Workshop*[2]	municipal public health bureaus.	self-management, health literacy and wellbeing		of Persons Attributable to the High-Risk Group of Cardiovascular Diseases";<br>2) Adults, who are assigned to persons at risk.		
8	Walking on the path of wellbeing<br>Italia: IT1<br>2012–12-2014	To increase physical activity levels through an evidence-based action.	Health promotion and disease prevention, physical activity and wellbeing.	• Integrated approach	People with sedentary behaviour, in particular patients with chronic diseases and those over 65 years old.	6> sectors<br>3 disciplines	Process and outcome
9	VESOTE project<br>Finland: FI2<br>2017–2018<br>*Workshop*[2]<br>*Interview*[1]	To adopt and reinforce effective and high-quality lifestyle counselling operational models.	Health promotion and disease prevention: overweight, physical activity, healthy food and better sleep without medication	• National program<br>• Health Service Delivery<br>• Integrated approach<br>• Training, capacity building	Physically inactive persons, persons suffering sleep problems, diabetics, coronary patients, overweight patients and obese patients	> 6 sectors<br>> 6 disciplines	Process and participatory research
10	The Strength in Old Age Programme<br>Finland: FI3<br>2005–ongoing	To launch research-based health exercise for independently living elder adults (75+) with decreased functional capacity.	Health promotion: physical activity, health literacy, wellbeing and reduction of health inequalities	• National program<br>• Policy action<br>• Integrated approach<br>• Training, capacity building<br>• Online intervention program	Community-living 75+ persons with decreased mobility and intersectoral collaboration group.	3-5 sectors<br>3 disciplines	Process and outcome
11	The Hygiene Week<br>Denmark: DK1<br>2009–2019 (every year)	To raise interest in population, in media and among decision makers to use hygiene to prevent infections - creating empowered, health literate citizens to (also) prevent AMR.	Health promotion and disease prevention: self-management and health literacy	• National program<br>• Community intervention<br>• Health Service Delivery<br>• Policy action<br>• Integrated approach<br>• Media campaign	General population	3-5 sectors<br>4-5 disciplines	Process

	Practice	Aim	Topic and Themes	Type	Target Group	Collaboration	Evaluation
12	The Andalusian Strategy of Local Action in Health Spain: ES1 2008–ongoing	To bring public health on the local agenda of all existing municipalities in Andalusia.	Health promotion and disease prevention: healthy aging, accident prevention, sexual and reproductive health, violence prevention, gender issues, environmental health and urban health.	• Community intervention<br>• Policy action<br>• Integrated approach<br>• Training, capacity building<br>• Intersectoral approach<br>• Participation<br>• Governance	General population of 778 municipalities of the Autonomous Community of Andalusia (Spain).	> 6 sectors<br>4-5 disciplines	Process and participatory
13	Gaining Health - making healthy choices<br><br>Italy: IT2 2007–ongoing	To reduce the impact of common risk factors on the population with cancer, cardiovascular diseases, diabetes and chronic respiratory diseases.	Health promotion and disease prevention: overweight, physical activity, alcohol prevention, smoking, wellbeing, reduction of health inequalities and nutrition	• National program<br>• Community intervention<br>• Policy action<br>• Integrated approach	Life course approach: addressing all ages and all public and private environments.	> 6 sectors<br>3 disciplines	Process and outcome
14	Living Healthy Croatia: CR1 2016–2022	To reduce the negative impact of behavioural, biomedical and sociomedical risk factors for the development and early onset of chronic NCDs and to inform, educate and raise awareness on the positive aspects of healthy lifestyles.	Health promotion and disease prevention: overweight, physical activity, alcohol prevention, smoking, health literacy, wellbeing and mental health/child depression	• National program<br>• Community intervention<br>• Integrated approach<br>• Training, capacity building	Life course approach: with a special focus on persons with heightened behavioural and biomedical risk factors.	>6 sectors<br>3 disciplines	Process, outcome and participatory
15	Coordinated strategy and action in health promotion for school health care	To implement a coordinated strategy and action in health promotion for the school health care service in Iceland.	Health promotion and disease prevention: overweight, physical activity, alcohol	• National program	School-aged children (6-15 years old) as well as school nurses, teachers and	2 sectors<br>3 disciplines	Process and participatory

	Practice	Aim	Topic and Themes	Type	Target Group	Collaboration	Evaluation
	Iceland: IS1<br>2006–ongoing		prevention, smoking, self-management, health literacy, wellbeing and reduction of health inequalities		other school personnel.		
16	The process towards a smoke-free Hungary – Tobacco control in practice<br>Hungary: HU1<br>2011–ongoing<br>*Interview*[1]	To reduce illness, disability, and death related to tobacco use and second-hand smoke exposure by raising awareness and health education.	Health promotion and disease prevention: smoking	• National program<br>• Policy action<br>• Case study	Children, young adults and adults.	2 sectors	Process and outcome
17	Living with Diabetes: Education and Weight Management<br>Malta: MT1<br>2015 -ongoing	To provide adult patients with diagnosed type 2 diabetes with the opportunity to undergo an educational and intensive weight management program delivered by a multidisciplinary team.	Health promotion and disease prevention: overweight, physical activity, self-management and health literacy	• National program	Overweight and obese patients who have type 2 diabetes.	-	Outcome
18	Roma health mediators<br>Serbia: S1<br>2009–ongoing	To improve health and quality of life of Roma population in Serbia.	Health promotion and disease prevention: health literacy, well-being and reduction of health inequalities	• National program<br>• Community intervention<br>• Health Service Delivery<br>• Training, capacity building	Roma ethnic minority population in Serbia.	3-5 sectors and < 2 disciplines-	Process
19	National programme for prevention of NCDs (noncommunicable diseases) - 2013–2020<br>Bulgaria: BG1	To promote population's health and improve quality of life by reducing premature mortality, morbidity and health outcomes due to major NCDs.	Health Promotion and disease prevention: overweight, physical activity, alcohol prevention, smoking, self-management, health literacy and	• National program<br>• Community intervention<br>• Health Service Delivery<br>• Policy action<br>• Integrated approach<br>• Training, capacity building	Life course approach: but especially focuses on women of reproductive age, workplaces, health professionals and	>6 sectors	Process, outcome and participatory research

International Journal of
Environmental Research and Public Health

	Practice	Aim	Topic and Themes	Type	Target Group	Collaboration	Evaluation
	2013–2020		main NCDs: Cardiovascular diseases, malignant neoplasms, diabetes, COPD		individuals with low socioeconomic status		
20	The Lombardy Workplace Health Promotion (WHP) Network Italy IT3 2014–ongoing *Interview*[1]	To join efforts of employers, employees and society, to improve health and welfare in the workplace.	Health promotion and disease prevention: physical activity, alcohol prevention, smoking, food, work-life balance and road safety	• Integrated approach<br>• Regional program	All company workers are involved (young adults, adults, male and female).	2 sectors	Process

[1] This practice has been interviewed for more in depth information. [2] This practice has been presented during the workshop

International Journal of
Environmental Research and Public Health

Table S2. Seven recommendations for Intersectoral Collaboration with the rationale and steps to implement the recommendation and examples of the practices which show the recommendation in practice.

1	**Connect collaborating goals with existing key policies, while actively advocating for political support.**<br>Why?<br>Political support is a prerequisite to get resources allocated for the implementation and the sustainability of health-promotion programs. In order to gain political support, collaboration goals should be aligned with key policies<br>How?<br>• Ensure that the planning documents contain the references to important policies<br>• Align with health system goals<br>• Make use of existing system changes<br>**Example**: Local Action in Health (RELAS), Andalusia, Spain (2008-ongoing)<br>The Autonomous Community of Andalusia (Spain) is carrying out the comprehensive strategy known as Local Action in Health (RELAS), which entails a thorough process to build alliances within the Andalusian municipalities, for instance among their mayors, government boards, stakeholders and, above all, their citizens. It is a common space for the contribution and cooperation of all stakeholders involved, and it represents the Andalusian public commitment to support intersectoral collaboration for health in all the territories.<br>In order to implement the Health in All Policies approach, a local work-plan (the Plan of Local Action in Health) is developed specific to each municipality. Each plan is conceived, built and carried out with the collaboration of all possible partners and taking into special consideration the participation of citizens. They all play an active role in all phases of the preparation of the Plan of Local Action in Health. This Plan of Local Action in Health is the basic implementation instrument, which is comprised of the planning, management, and coordination of all the actions that are relevant to public health in the municipality.<br>It is recommended to elevate this Plan of Local Action to the Municipal Plenary, as this is an exercise of government and consensus among all the political forces represented in the municipality, and it would further guarantee the appointment of the necessary resources for an established period of 4-5 years.
2	Define a shared vision of the problem to be solved aligned with organizational goals<br>Why?<br>Commitment of all partners is crucial for successful collaboration. Agreeing on the problem to be solved and defining a shared vision of how to solve the problem helps to create this commitment and results. Furthermore, such a discussion allows professionals from different organizations, and possibly sectors, to develop a common language to talk about the main issues and potential solutions.<br>How?<br>• Appeal to a shared sense of urgency to solve a problem or to shared interests<br>• Agree on intersectoral collaboration as one of the solutions of the problem<br>• Achieve actual mutual understanding of norms, values, and roles and create trust<br>• Use a visionary leader who is accepted by all parties<br>• Engage an experienced facilitator/coordinator<br>**Example:** Healthy Overvecht, Integrated medical and social care, the Netherlands (2006-ongoing)<br>The collaboration was originally developed in a deprived neighbourhood in Utrecht (Utrecht Overvecht), in response to the needs of primary health care professionals in this neighbourhood (e.g., general practitioners, physiotherapists, midwives, Youth Health Care Services, Municipal Public Health Services team, and district nurses). They felt a great deal of work pressure and indicated that the situation was not sustainable. There was a shared feeling of urgency among professionals, the municipality, and other organisations to solve this problem together. They defined a shared vision of how to solve these problems. For instance, they agreed to all use the same interview model (4D-model) for their patients. This is a model to methodically map the patient's problems. While filling out the 4D-model, the professional looks together with the patient at what is going well in the domains of body, mind, social and relations/network, and what problems there are. They also created direct lines of communication across sectors. Professionals from the social domain (e.g., social workers and neighbourhood teams), who now also use the same interview model for their clients, share information with primary health care professionals, taking advantage of the substantial overlap in clients/patients. This makes their work more efficient and alleviates work load. At present, the collaboration has a 'quadruple aim': improving the perceived health of patients, the efficiency of care, the quality of care, and the job satisfaction of the professionals involved. Due to the success of Healthy Overvecht, it is now being piloted in twelve other deprived neighbourhoods in the cities of Utrecht, Rotterdam, Amsterdam and the Hague through December 2020.

Create an effective mix of different partners with diverse backgrounds and skills

Why?

To be able to reach the target group effectively, all relevant parties that could influence the health behavior of the target group should be involved in the collaboration.

How?

- Identify and involve strategic partners with access to and/or experiences with the target group
- Capitalize on existing partners and available collaboration networks
- Allow ample time for building new relationships
- Involve representatives of the target group and community from the start
- Use standard methods for stakeholder mapping

Example: The Lombardy Workplace Health Promotion (WHP) Network, Italy (2011-ongoing)

3

The Lombardy WHP Network is a member of the European Network for Workplace Health Promotion and it builds multi-stakeholders partnerships and collaboration at horizontal and vertical levels, mixing up public and private sectors. It aims to join efforts of employers, employees and society, to improve health and welfare in the workplace. The main partners to initiate the formal collaboration in order to create sustainable actions were: Sodalitas Foundation [National Partner Organization of European Business Network for Corporate Sustainability and Responsibility (CSR Europe], Confindustria Lombardia (associations of companies), trade unions and the regional healthcare system at its different organizational and structural levels. Also, the community and target groups were involved from the start in the collaboration process and the programme implementation at the workplaces. A manual for companies that join the network recommends involving employees and other key roles from the beginning, to plan their programmes and select good practices based on their specific needs. Other partners can be involved at different levels: associations of professionals, non-profit organizations (with special reference to social/sport activities promotion associations or with expertise on specific health issues such as smoking cessation), municipalities, scientific societies and universities. Due to the collaboration project, the healthcare system gained more skills in interacting with other sectors of society (e.g., companies), recognising its own limits in influencing certain multi-faceted determinants of health.

Build bridges between sectors and disciplines through effective leadership

Why?

Leadership is essential and closely tied to strong working relationships and a transparent process for collaboration. Effective leadership fosters trust and good working relationships between collaboration partners.

How?

- Identify a local champion who can be the leader or can support the leader
- Use different types of leaders or leadership for different phases of the collaboration
- Recruit a dedicated person with leaderships and coordination qualities:
- who understands the language of 'others'
- with good project and process management skills
- who uses information systems and technologies to ensure effective communication and information exchange

Example: The process towards a smoke-free Hungary – Tobacco control in practice (2011-ongoing)

4

The Prime Minister of Hungary is dedicated to the anti-tobacco cause. He was adamant that signing international legislations is not enough, it also need to be implemented. Since then, guidelines, protocols and recommendations were disseminated to provide guidance on tobacco control, such as the Framework Convention on Tobacco Control of the WHO. There is also a dedicated person, who possesses the necessary information and expertise in the field to act as a coordinator. This person, who despite the high turnover of professionals in the field, has been present for 30 years and has been the one constant in tobacco control. He serves as the coordinator between the supporting departments, ministries and non-governmental organizations.

He has been working as a health promotion programme manager of the national health programme and co-ordinates activities in Hungary in connection with smoking prevention and cessation. As Head of the Hungarian Focal Point for Tobacco Control, his main tasks include: making plans in the short, medium and long term related to tobacco control, making professional, methodological guidelines, and making recommendations on public health and health development. He is responsible for elaborating professional programmes' methodologies, creating and maintaining a database of laws and provisions, and of instructions for their use; supervising the collection of social, economic and health indicators related to tobacco consumption; conducting research; fulfilling organisational tasks and coordination.

In addition, he is the creator and leader of the Smoking Prevention Programme for Kindergarten Children (age 3-6), which has been introduced in one third of all the kindergartens in Hungary, and the Smoking Prevention Program for Primary School Children (age 6-10), which has been running in one quarter of the elementary schools in the country. In connection with these programmes, he leads the activities of producing and developing special health education

materials. He is also one of the leaders of the professional co-ordination of the activity of the national network of the National Public Health and Medical Officer's Service in connection with smoking.

This collaboration has strengthened other health promotion activities beyond tobacco control.

Keep collaboration partners in all sectors engaged

Why?

Crucial for the success of the collaboration is keeping the partners engaged by informing, motivating, and entrusting them, thus sustaining commitment of all partners.

How?

- Formalize the collaboration by making clear agreements about roles and responsibilities of the partners
- Create a win-win situation for partners in the collaboration (mutual and joint benefits)
- Form designated communication liaisons, e.g., to provide information to participants of the collaboration, arrange meetings, manage a website, and/or create regular newsletters
- Give professionals ownership, via a bottom-up approach
- Motivate the professionals involved, e.g., by offering feedback on progress towards shared vision
- Celebrate smaller short-term advancements while aiming for long-term, sustainable success
- Organize face-to-face meetings when possible to allow people from different sectors and disciplines to get to know each other also on an informal and personal level

5 **Example:** VESOTE project, Finland (2017-2018)

The VESOTE programme reinforces and develops effective and target-based lifestyle guidance in social and health care. The development activities emphasise physical activity, nutrition and sleep. The final goal of the programme is for Finns to be more physically active, sit less, eat a varied and healthy diet and sleep better.

The municipalities of Northern Ostrobothnia signed a joint plan for strengthening cooperation between social and health care actors and between social and health care and other actors. The primary target group was those of working age - obese adults and arterial patients - as well as those at high risk for developing arterial disease. The project created new cooperation groups and strengthened the activities of existing ones. Active communication was a success factor. At the beginning of the project, a communication plan was developed, which received the approval of the development manager in the hospital district. Communication was goal-oriented. Project leaders sought out the tools and the help of communication experts. Visibility in regional media was obtained; in fact, there were several different media channels including a local magazine and Facebook. The project resulted in new perspectives and expertise for health professionals. It is hoped that cooperation will continue in the future. Many different actors have promised to participate in the long-lasting partnership, and their will to act has strengthened. The initiative to set up a lifelong learning Centre has been established.

Use a planned/systematic approach suitable for all partners

Why?

Using a systematic approach based on scientific evidence and on experiences from the past will improve the implementation of the collaboration in each sector. Moreover, this systematic approach should allow all partners to combine their health-promotion efforts and enhance the effectiveness of the program.

How?

- Identify a theoretical framework or model that can be used by different sectors
- Identify a theoretical framework or model that can be adapted to local context
- Strengthen the collaboration as iterative and adaptive processes
- Share and learn from experiences
- Involve experts and others with experience in similar efforts
- • Replicate, and adapt if necessary, best practices that have been shown to result in successful outcomes

6 **Example:** Young People at a Healthy Weight (JOGG), the Netherlands (2014-ongoing)

JOGG is a program based on a previous project in France (EPODE), but has evolved since then. The objective of JOGG is to allow children to grow up in good health using an integrated approach at both the national and local level to target overweight. JOGG advocates a local approach in which parents and health professionals, shopkeepers, companies, schools and local authorities join forces to ensure that young people remain at a healthy weight. The Dutch JOGG approach consists of five pillars: political and governmental support; cooperation between the private and public sector (public private partnership); social marketing; scientific coaching and evaluation; linking prevention and health care. Although the programme has some pre-determined (five pillars) elements, it can be adapted to the local context. Over 140 municipalities and 30 social organizations and companies have joined JOGG. JOGG is a learning organisation and maintains contact with its partners to discuss the progress of the collaboration. In addition, an independent institute monitors what efforts JOGG has undertaken to commit towards their objectives.

Ensure there are sufficient resources to sustain the collaboration

Why?

7 To establish a sustainable collaboration, it is important that resources, such as dedicated time, qualified personnel, and funding, are and remain available. The distribution of these resources should be transparent and fair to all partners.

How?

- Describe needed and obtained resources to facilitate a transparent distribution among the partners
- Allocate (working hours of) personnel to collaboration
- Provide training to managers and professionals
- Acquire or build upon structural resources (e.g., human resources or funding)
- Communicate about the cost-saving or effective results

Example: Tobacco Cessation Services for Patients with Mental Health Disorders and Substance Abuse (Finland 2017-2018)

Filha had an initial project idea, and, when the appropriate funding mechanism became available (a government programme to disseminate good practices), it enabled the project to start. The project sought to identify what had been done in hospital districts on the subject of tobacco cessation among mentally ill patients and patients with substance abuse problems in order to develop regionally-appropriate activities. The ultimate goal was to improve to quit smoking for mentally ill smokers and patients with a history of substance abuse received to quit smoking. Hospital districts developed their own models where collaboration has occurred between primary and secondary care, as well as with NGO's: The part-time regional facilitator, who was especially assigned to this project in every participating hospital district, discussed with and trained the staff in different departments together with Filha. He also collaborated with local NGO's, who had contacts with experts by experience. Courses were organised in the hospital districts and a 2-hour on-line course was available. It was agreed that staff would attend these courses. The regional facilitator visited the different departments in secondary as well as primary care in the hospital district and convinced the workers to attend the courses. He discussed practical issues with the workers around providing tobacco cessation services and helped them resolve issues that arose. Some hospital districts recognise the value of this project and are allocating their own resources to fund a regional facilitor now the initial program has ended.
